# The Challenge of Appropriate Identification and Treatment of Starvation, Sarcopenia, and Cachexia: A Survey of Australian Dietitians

**DOI:** 10.1155/2011/603161

**Published:** 2011-12-25

**Authors:** Alison Yaxley, Michelle D. Miller

**Affiliations:** ^1^Rehabilitation and Aged Care, Clinical Effectiveness, Flinders University, P.O. Box 2100, Adelaide, SA 5000, Australia; ^2^Nutrition and Dietetics, Clinical and Molecular Medicine, Flinders University, P.O. Box 2100, Adelaide, SA 5000, Australia

## Abstract

Malnutrition is an umbrella term that includes starvation, sarcopenia, and cachexia; however, differentiating between these terms is infrequent in clinical practice. Given that the effectiveness of treatment depends on the aetiology of unintentional weight loss, it is important that clinicians are aware of the defining characteristics. The aim of this study was to determine whether Australian dietitians understand and use the terms starvation, sarcopenia, and cachexia and provide targeted treatment strategies accordingly. Members of the Dietitians Association of Australia were surveyed to gain information on practices and attitudes to diagnosis and treatment of adult malnutrition. In addition, three case studies were provided to examine understanding of starvation, sarcopenia, and cachexia. 221 dietitians accessed the survey. 81 respondents (43%) indicated the use of at least one alternate term (starvation, sarcopenia, and/or cachexia). Muscle wasting was the most commonly used diagnostic criterion. High-energy high-protein diet was the most common therapy prescribed. Correct diagnoses for case studies were recorded by 6% of respondents for starvation, 46% for sarcopenia, and 21% for cachexia. There is a need for increased awareness of the existence of starvation, sarcopenia, and cachexia amongst Australian dietitians and research into appropriate methods of identification and treatment for each condition.

## 1. Introduction

Recent literature has refined the umbrella term malnutrition to include such conditions as starvation, sarcopenia, and cachexia [1–3]. In recent years, several consensus definitions for the diagnosis of sarcopenia [1, 4] and cachexia [1, 5] have been published, however, there is no indication that further work has been conducted to validate any of these definitions. Consequently, there is still confusion over the diagnosis of these conditions as there are no universally shared criteria. However, indications are that simple starvation is the loss of fat and fat-free mass which occurs purely as a result of protein-energy deficiency; sarcopenia is the loss of muscle mass and muscle strength which occurs with ageing; cachexia is severe wasting, predominantly of fat-free mass, driven by inflammation [4–7].

Guidelines used in practice, including the Dietitians Association of Australia (DAA) endorsed “Evidence-based practice guidelines for the nutritional management of malnutrition in adult patients across the continuum of care,” invariably take a simplistic approach to malnutrition through focusing on protein-energy malnutrition, or starvation, alone [2, 7, 8]. The implications of the scarcity of recommendations for identification and treatment of alternate malnutrition conditions (sarcopenia and cachexia) are potential misdiagnosis and treatment, and ultimately suboptimal health outcomes.

While increased energy and protein intake may be the principal, and most appropriate, intervention for treatment of starvation, it is becoming clear that conditions such as sarcopenia and cachexia cannot be reversed by increased dietary intake alone [9]. Investigation into pharmacological therapies for sarcopenia has not provided strong evidence for efficacy, however, there is support for resistance training, in addition to adequate energy and protein intake, as a safe and effective therapy [10]. Considerable research has been conducted in an effort to find an effective therapy for cachexia. Potential interventions include megestrol acetate [11], ghrelin [12], and fish oil [13], however, success has been limited and research is continuing.

In the battle to combat malnutrition dietitians, are in the frontline yet the success of their practice could be limited unless they are armed with the correct knowledge in terms of identification and appropriate treatment strategies. The aim of this project was to determine whether dietitians understand and use the terms starvation, sarcopenia, and cachexia and provide targeted treatment strategies accordingly.

## 2. Materials and Methods

An anonymous cross-sectional survey of members of the DAA was undertaken to gain information on their attitudes and practices regarding the diagnosis and treatment of malnutrition in adults. All members are required to have graduated from a tertiary program designed to achieve entry level competencies as prescribed by the DAA. Members of the DAA, including student members, were invited to participate via weekly email distribution from DAA national office, in August 2010. The email notified of the presence of a link to an online questionnaire and provided a brief introduction to the survey to enable prospective participants to gauge their level of interest in the project. Upon entering the web-based survey, participants were provided with further background. At the time of the survey, the DAA website indicated a potential pool of approximately 4000 members, and all were eligible to participate in the survey. Researchers were blinded to respondents' identities. The protocol for this research project was approved by the Social and Behavioural Research Ethics Committee of Flinders University, Adelaide, South Australia, and conforms to the provisions of the Declaration of Helsinki (as revised in Edinburgh 2008). Informed consent was implied upon completion of the survey. This study was registered on the Australian New Zealand Clinical Trials Registry (ACTRN12610000403033).

Following a review of the current literature on identification and classification of categories of malnutrition, including relevant consensus definitions [1, 4, 5], a questionnaire was drafted by the authors using an online survey facility (SurveyMonkey, Palo Alto, Calif, USA). The tool was piloted on a small group of clinical dietitians and subsequently amended to reflect their comments where necessary. The final web-based questionnaire comprised a maximum of 42 questions (some conditional release), depending on responses given (see Table 1), and was available online from August-September 2010. Participants were asked to complete the survey by selecting appropriate answers from random-ordered lists provided and by giving further comment where necessary. A copy of the survey is available from the corresponding author on request.

Demographic information collected included age, gender, years of practice, and location. In order to ascertain current practice and use of terminology, respondents were asked to provide information on criteria for diagnosis, exposure to malnourished patients, use of the terms starvation, sarcopenia, and/or cachexia and criteria for diagnosis of any categories used. Three case studies were provided; one describing a patient in each of the three categories of malnutrition of interest, according to current literature [1, 5, 9], and respondents were asked to provide a diagnosis based on information given. Treatment regimes were investigated in a question asking respondents to rank ten possible interventions for malnutrition, from one to ten. Further investigation was then conducted into the use of specific interventions (fish oil, appetite stimulants, and resistance training), which have been investigated as potential therapies for sarcopenia and cachexia [10, 14, 15].

Survey responses were analysed using SurveyMonkey, and IBM SPSS Statistics, version 19.0.0 [16]. Descriptive statistics were reported as frequencies (*n*) and percentages (%). Chi-squared tests were used to compare case study responses for selected subgroups of study participants. Statistical significance was set at *P* < 0.05.

## 3. Results

Two hundred and twenty-one DAA members, including ten student members, accessed the web-based survey during the data collection period, representing a response rate of approximately 5.5%. Of those, 209 (95%) provided full demographic details and 205 (93%) continued to the main part of the survey. In total, 169 (76%) respondents completed the entire survey.

Respondent characteristics are reported in Table 2. Responses were received from all states and territories in Australia, with the majority from Victoria (30%) and New South Wales (30%). Almost half of respondents worked in the public hospital system (49%) and one-quarter reported some work in private practice (25%). Ninety percent reported working with adult patients.

Around 40% of survey participants reported seeing 2–5 malnourished adults each week, with some reporting that they consulted ≥10, particularly in the aged care setting. With regard to terminology to describe categories of malnutrition, 81 respondents (43%) indicated that they used at least one of the terms starvation, sarcopenia, and/or cachexia. Of those, most used cachexia (89%), followed by starvation (31%) and sarcopenia (19%). Six (7%) individuals reported use of all three terms in their practice.

The most common criteria for diagnosis of malnutrition were reported to be muscle wasting (86%), loss of subcutaneous fat (81%), weight loss (77%), decreased appetite (76%), BMI < 18.5 kg/m^2^ (<22 kg/m^2^ for those aged 65+ years) (67%), and anorexia (65%). For respondents reporting that they used the term starvation, the top three criteria used to diagnose this condition were muscle wasting (38%), anorexia (36%), and low BMI (36%). For respondents reporting that they used the term sarcopenia, the top three criteria used to diagnose this condition were muscle wasting (31%), loss of muscle strength (28%), and weight loss (16%). For respondents reporting that they used the term cachexia, the top three criteria used to diagnose this condition were muscle wasting (76%), loss of subcutaneous fat (74%), and anorexia (69%).

When responding to the case study questions, the majority of respondents (77%, 63%) used the term malnutrition to refer to what were clear cases of starvation and cachexia, respectively. The correct diagnosis of starvation was recorded by 6% of respondents; 46% of respondents correctly identified the sarcopenia case; 21% correctly identified cachexia. When case study responses were examined according to years of dietetic experience, more respondents with 5 or less years of experience correctly identified the condition in each case study, however these differences were not statistically significant (see Table 3).

When participants were asked to rank, from 1 to 10, a range of possible interventions to treat malnutrition, according to their own criteria, the most frequently selected therapies were high-energy high-protein diet (75% of respondents), high-energy high-protein snacks (48%), oral nutritional supplements (42%), and enteral feeds (32%). Three selected therapies were further investigated: 21% of respondents have recommended appetite stimulants to patients, 45% have recommended fish oil, and 57% have recommended resistance training. However, survey respondents reported that very few patients received such a recommendation. Fifty percent of those recommending appetite stimulants indicated that this recommendation was for less than 5% of patients. Similarly, 64% of those recommending fish oil, and 46% of those recommending resistance training, did so to less than 5% of their patients.

Most commonly, respondents referred patients for prescription appetite stimulants (56%) and referred to a physiotherapist for resistance training (71%) but made their own recommendations for fish oil. Fish oil was proposed most often for high cholesterol (75%) and rheumatoid arthritis (71%), with most respondents advising a daily dosage of 1 g of omega-3 fatty acids per day. Twenty six percent of study participants recommended fish oil as an intervention for malnutrition.

## 4. Discussion

The results from this cross-sectional survey provide insight into the inconsistency in the understanding and use of the terms starvation, sarcopenia, and cachexia amongst Australian dietitians. Furthermore, it appears that treatment strategies are generally focused on improving energy and protein intake, a strategy that alone is known to be largely ineffective in the treatment of cachexia and sarcopenia [7, 10].

Alternative terminology for different categories of malnutrition was not widely used amongst Australian dietitians, with less than half of respondents using any of the terms starvation, sarcopenia, and/or cachexia. While the recently released DAA endorsed “Evidence based practice guidelines for the nutritional management of malnutrition in adult patients across the continuum of care” are explicit in their reference to protein-energy under nutrition only [8]; other commonly used guidelines and tools do not differentiate between categories of malnutrition [17–19], therefore they do not provide dietitians with the necessary guidance to correctly diagnose different malnutrition conditions.

Those who use the terms starvation, sarcopenia and/or cachexia do not appear to be consistent in using the most accepted criteria as part of their diagnosis. Simple starvation is unintentional weight loss, with predominant loss of fat mass, purely as a result of inadequate protein and energy intake [3], yet 38% of respondents indicated that muscle wasting was the key criterion for diagnosis, more than reduced BMI or weight loss. The key characteristics of cachexia are elevated inflammatory markers and reduced body cell mass [9], so while 76% of respondents indicated that muscle wasting was their key criterion for diagnosis, only 24% would consider elevated inflammatory markers. Sarcopenia is characterised by reduced muscle mass, strength, and function [1, 4], and 76% of survey participants indicated that muscle wasting was again the key criterion, but 50% of respondents indicated that muscle strength was a consideration hence this appears to indicate a clearer understanding of sarcopenia amongst Australian dietitians.

Ability to identify the three conditions from case study information was poor, with 6% of respondents able to correctly diagnose starvation, 46% sarcopenia, and 21% cachexia. This gives further indication that dietitians are more familiar with sarcopenia than either starvation or cachexia and suggests that there is a need to increase the awareness among dietitians of the existence of separate categories of malnutrition and to educate on alternate diagnoses. However, for each case study, more newer graduates (5 or less years of experience) than those with 6 or more years of experience, correctly identified the different categories of malnutrition. Although this did not reach statistical significance it does suggest that these categories may receive more attention in current university curriculums. It is noteworthy that 63% of respondents to the cachexia case study indicated a diagnosis of malnutrition. While this is technically not incorrect as all cachectic patients are malnourished, the case study provided information on inflammatory status which should have alerted dietitians to the presence of cachexia [1].

The inconsistency identified in the understanding and application of the terms sarcopenia, starvation, and cachexia extended to the interventions used to treat patients/clients. Therapy for malnutrition was predominantly centred on increased energy and protein intake with most dietitians prescribing high-energy high-protein diet, snacks and/or enteral feeds. However, the literature indicates that the defining characteristic of starvation is that it is reversed by increased protein and energy intake, unlike sarcopenia or cachexia, therefore, provision of standard interventions, regardless of the aetiology of malnutrition is likely to address starvation alone.

There are some limitations which should be considered in the interpretation of the data from this survey. Participants were from Australia and it should be acknowledged that the findings may differ elsewhere and therefore a repeat survey of dietitians from other countries would be valuable. The sample size may be considered small, however the response rate was similar to the expected level of response to surveys previously delivered by the organisation (DAA national office, personal communication, 2010). It is also noteworthy that the majority of respondents were young and had been practicing for a short time and worked in metropolitan public hospitals. This profile reflects the workforce statistics for dietitians in Australia (DAA national office, personal communication, 2010). Finally, the diagnostic criteria used in the questionnaire were based on consensus definitions which have not yet been validated however they were developed by leading experts in the field and are therefore likely to be proved to be valid and reliable in future work.

Further investigation into three interventions previously researched for use in different categories of malnutrition revealed that appetite stimulants, fish oil, and resistance training are not widely recommended to malnourished patients. Recent research has shown that these interventions can be helpful in conjunction with adequate energy and protein intake to address sarcopenia and cachexia, which are unlikely to resolve purely with increased nutritional intake [10, 14, 15]. However, currently used guidelines for treatment of malnutrition do not advocate targeted therapies for malnutrition [8, 17, 19] thus do not provide dietitians with the correct advice to deliver optimal health outcomes to patients.

In conclusion, the results of this study indicate a need for increased awareness of starvation, sarcopenia, cachexia, and appropriate methods of identification and treatment. More work is warranted to explore how dietitians might expand existing or collaborate to develop new screening and/or assessment tools to assist in differentiating the three distinct conditions, contribute to the evidence base for appropriate treatment strategies, and determine the cost effectiveness of this change in practice.

## Figures and Tables

**Table 1:** Details of online questionnaire used to survey DAA members on perspectives on malnutrition.

Topic	Maximum number of questions	Response format	Examples of content
Demographic questions	11	Yes/no tick boxes; 2–8 tick boxes per question; space for comments	Age; gender; number of years of experience; employment status
Diagnosis of malnutrition	10	Yes/no tick boxes; 2–16 tick boxes per question; space for comments	ICD criteria for malnutrition; choice of markers for diagnosis of malnutrition; use of the terms starvation, sarcopenia and/or cachexia; choice of markers for diagnosis of starvation, sarcopenia and/or cachexia, if used
Case studies	3	4 tick boxes per question; space for comments	One case study for each of starvation, sarcopenia and cachexia
Treatment of malnutrition	12	Choice of 10 possible interventions to be ranked 1–10; yes/no tick boxes; 5-6 tick boxes per question; space for comments	Choice of possible interventions; further questions on specific interventions: appetite stimulants, fish oil, and resistance training
Barriers to effective treatment of malnutrition	1	Open-ended; space for comment	
Guidelines for treatment of malnutrition	3	Yes/no tick boxes; space for comment	DAA guidelines; other guidelines used

Total	40^†^		

^†^A further two questions asked the participant about their desire to continue in order to ascertain the correct path through the questionnaire.

DAA: Dietitians Association of Australia.

**Table 2:** Characteristics of 221 respondents to a web-based survey of current attitudes and practices of members of the DAA, in regards to diagnosis and current dietary management of malnutrition.

	Female (*n* = 215)	Male (*n* = 6)
Gender (*n* = 221)	97.3%	2.7%
Age		
(i) 21–30 years	51.6%	33.3%
(ii) 31–40 years	26.0%	33.3%
(iii) 41–50 years	14.0%	16.7%
(iv) 51–60 years	6.5%	16.7%
(v) >60 years	1.9%	
Employment status		
(i) Full time	65.1%	83.3%
(ii) Part time (<20 hours per week)	16.3%	16.7%
(iii) Student dietitian	4.7%	
(iv) Locum	2.3%	
(v) Further study	0.9%	
(vi) Unrelated industry	0.9%	
(vii) Not working	1.9%	
(viii) Retired	0.5%	
(ix) Other	7.4%	
Dietetic experience		
(i) <1 year	8.4%	
(ii) 1–5 years	35.3%	66.7%
(iii) 6–10 years	19.5%	16.7%
(iv) 11–20 years	18.1%	16.7%
(v) >20 years	14.0%	
Work location		
(i) Metropolitan/urban	63.3%	50.0%
(ii) Regional/rural/remote	27.4%	33.3%
(iii) Both of the above	4.2%	16.7%
Primary practice setting		
(i) Public hospital	47.8%	83.3%
(ii) Community health	21.5%	16.7%
(iii) Private hospital	4.4%	
(iv) Private practice^†^	10.2%	
(v) Other	16.1%	

^†^A further 15.2% of respondents reported part-time private practice.

DAA: Dietitians Association of Australia.

**Table 3:** Case study responses reported by respondents to a web-based survey of current attitudes and practices of members of the DAA, in regards to diagnosis and current dietary management of malnutrition, according to years of dietetic experience (*n*/%).

	<1–5 years	6+ years
Case study 1: Cachexia		
(i) Malnutrition	56 (62%)	61 (66%)
(ii) Starvation	0 (0%)	0 (0%)
(iii) Sarcopenia	5 (6%)	4 (4%)
(iv) Cachexia	**21 (23%)**	17 (18.3)
Case study 2: Starvation		
(i) Malnutrition	69 (78%)	72 (78%)
(ii) Starvation	**8 (9%) **	3 (3%)
(iii) Sarcopenia	2 (2%)	5 (5%)
(iv) Cachexia	6 (7%)	7 (8%)
Case study 3: Sarcopenia		
(i) Malnutrition	28 (33%)	31 (34%)
(ii) Starvation	4 (5%)	5 (6%)
(iii) Sarcopenia	**44 (52%) **	34 (37%)
(iv) Cachexia	4 (5%)	11 (12%)

*n*: number.

## References

[1] Muscaritoli M, Anker SD, Argilés J (2010). Consensus definition of sarcopenia, cachexia and pre-cachexia: joint document elaborated by Special Interest Groups (SIG) “ cachexia-anorexia in chronic wasting diseases” and ‘nutrition in geriatrics’. *Clinical Nutrition*.

[2] Soeters PB, Schols AM (2009). Advances in understanding and assessing malnutrition. *Current Opinion in Clinical Nutrition and Metabolic Care*.

[3] Jensen GL, Mirtallo J, Compher C (2010). Adult starvation and disease-related malnutrition: a proposal for etiology-based diagnosis in the clinical practice setting from the international consensus guideline committee. *Journal of Parenteral and Enteral Nutrition*.

[4] Cruz-Jentoft AJ, Baeyens JP, Bauer JM (2010). Sarcopenia: European consensus on definition and diagnosis: Report of the European Working Group on Sarcopenia in Older People. *Age Ageing*.

[5] Evans WJ, Morley JE, Argilés J (2008). Cachexia: a new definition. *Clinical Nutrition*.

[6] Collins N (2010). Sarcopenia, cachexia, and starvation. *Ostomy Wound Manage*.

[7] Messinger-Rapport BJ, Thomas DR, Gammack JK, Morley JE (2009). Clinical update on nursing home medicine: 2009. *Journal of the American Medical Directors Association*.

[8] Watterson C, Fraser A, Banks M (2009). Evidence based practice guidelines for the nutritional management of malnutrition in adult patients across the continuum of care. *Nutrition and Dietetics*.

[9] Evans WJ (2010). Skeletal muscle loss: cachexia, sarcopenia, and inactivity. *American Journal of Clinical Nutrition*.

[10] Jones TE, Stephenson KW, King JG, Knight KR, Marshall TL, Scott WB (2009). Sarcopenia–mechanisms and treatments. *Journal of Geriatric Physical Therapy*.

[11] Yeh SS, Lovitt S, Schuster MW (2009). Usage of megestrol acetate in the treatment of anorexia-cachexia syndrome in the elderly. *Journal of Nutrition, Health and Aging*.

[12] DeBoer MD (2008). Emergence of ghrelin as a treatment for cachexia syndromes. *Nutrition*.

[13] Colomer R, Moreno-Nogueira JM, García-Luna PP (2007). N-3 fatty acids, cancer and cachexia: a systematic review of the literature. *British Journal of Nutrition*.

[14] Taylor LA, Pletschen L, Arends J, Unger C, Massing U (2010). Marine phospholipids-A promising new dietary approach to tumor-associated weight loss. *Supportive Care in Cancer*.

[15] Fox CB, Treadway AK, Blaszczyk AT, Sleeper RB (2009). ReviewsOf therapeutics megestrol acetate and mirtazapine for the treatment of unplanned weight loss in the elderly. *Pharmacotherapy*.

[16] IBM Corporation

[17] Lochs H, Allison SP, Meier R (2006). Introductory to the ESPEN guidelines on enteral nutrition: terminology, definitions and general topics. *Clinical Nutrition*.

[18] Kaiser MJ, Bauer JM, Ramsch C (2009). Validation of the Mini Nutritional Assessment short-form (MNA-SF): a practical tool for identification of nutritional status. *Journal of Nutrition, Health and Aging*.

[19] Mueller C, Compher C, Ellen DM (2011). A.S.P.E.N. clinical guidelines: nutrition screening, assessment, and intervention in adults. *Journal of Parenteral and Enteral Nutrition*.

